# The Interplay Between Gut Microbiota and miRNAs in Cardiovascular Diseases

**DOI:** 10.3389/fcvm.2022.856901

**Published:** 2022-03-16

**Authors:** Ruxandra Florentina Ionescu, Robert Mihai Enache, Sanda Maria Cretoiu, Dragos Cretoiu

**Affiliations:** ^1^Department of Cardiology I, Central Military Emergency Hospital “Dr. Carol Davila,” Bucharest, Romania; ^2^Department of Radiology and Medical Imaging, Fundeni Clinical Institute, Bucharest, Romania; ^3^Department of Morphological Sciences, Cell and Molecular Biology and Histology, Carol Davila University of Medicine and Pharmacy, Bucharest, Romania; ^4^Fetal Medicine Excellence Research Center, Alessandrescu-Rusescu National Institute for Mother and Child Health, Bucharest, Romania

**Keywords:** gut microbiota, miRNA, gut microbiome, trimethylamine oxide (TMAO), cardiovascular diseases

## Abstract

The human microbiota contains microorganisms found on the skin, mucosal surfaces and in other tissues. The major component, the gut microbiota, can be influenced by diet, genetics, and environmental factors. Any change in its composition results in pathophysiological changes that can further influence the evolution of different conditions, including cardiovascular diseases (CVDs). The microbiome is a complex ecosystem and can be considered the metagenome of the microbiota. MicroRNAs (miRNAs) are speculated to interact with the intestinal microbiota for modulating gene expressions of the host. miRNAs represent a category of small non-coding RNAs, consisting of approximately 22 nucleotides, which can regulate gene expression at post-transcriptional level, by influencing the degradation of mRNA and modifying protein amounts. miRNAs display a multitude of roles, being able to influence the pathogenesis and progression of various diseases. Circulating miRNAs are stable against degradation, due to their enclosure into extracellular vesicles (EVs). This review aims to assess the current knowledge of the possible interactions between gut microbiota, miRNAs, and CVDs. As more scientific research is conducted, it can be speculated that personalized patient care in the future may include the management of gut microbiota composition and the targeted treatment against certain expression of miRNAs.

## Introduction

Gut microbiota, represented by bacteria, viruses, fungi, archaea and protozoa, accounts for approximately 95% of the human microbiota (1, 2). The structure of the gut microbiota is influenced by host genetics and environmental factors. Recent studies have shown that gut microbiota is not just a passive participant in our health, but it can also actively regulate our functions *via* genes, proteins and metabolites (2–4). Perturbation of the gut microbiota, dysbiosis, can lead to several diseases, not only gastrointestinal conditions, but also disorders of the lung, brain, heart and immune system (5–7). Dysbiosis has an important role in CVDs, especially by activating a proinflammatory status in the body and favoring the atherosclerotic process (8, 9). Gut microbiota can metabolize L-carnitine, choline and phosphatidylcholine and produce vasculotoxic metabolites, such as trimethylamine-N-oxide (TMAO), which is associated with the atherosclerotic process (10). A continuous low-grade inflammation status in the gut can facilitate the transit of bacteria and their metabolites into the bloodstream, thus maintaining chronic inflammation and favoring the development of atherosclerotic plaques, increasing the risk of coronary heart disease, stroke, and other acute complications (9, 11).

miRNAs are regulators in the host gene expression processes and are linked to specific bacteria (12). They enter bacterial cells through endocytosis, influencing gut microbiota (13) by stimulating or inhibiting the expansion of certain bacterial species (14, 15). In the miRNAs-gut microbiota connection, miRNAs have effects on proliferation and differentiation of intestinal cells, architecture and barrier function of the gut (16). Some studies have shown that the gut microbiota can have an impact on miRNAs, by modulating the expression of genes in the host (12, 17). Moreover, the link between miRNAs and gut microbiota has been underlined by studies showing that EVs produced by gut microbiota include various RNA species, including miRNAs, which may influence gene expression of the host cells (13, 18, 19).

In recent years, the association between circulating miRNAs and CVDs has been widely investigated to discover new possible diagnostic markers (20, 21). In CVDs, miRNAs-gut microbiota relationship is involved especially in the regulation of epithelial dysfunction (7). Gut microbiota can influence RNA genes affiliated with miRNAs classes, like vascular miR-204, miR-10B or miR-181 (22), lowering or increasing the risk of developing atherosclerosis (23–25). Due to recent discoveries about the involvement of miRNAs-gut microbiota association in CVDs and the characterization of miRNAs as new possible diagnostic markers, studies have highlighted the importance of new therapeutic strategies (26).

Dietary interventions represent an important therapy, especially the use of plant-derived miRNAs to modify the gut microbiota (27). For example, studies on mice have shown that ginger-derived exosomes contain miRNAs that can influence the gut microbiota by increasing the metabolism of fatty acids. Wang et al. observed an increase in species of the Bifidobacterium genus and SCFA-producing bacteria, reducing body weight, liver steatosis, low-grade inflammation and insulin resistance. This study underlined the possibility of using ginger supplementation as a dietary intervention in CVD (27, 28). The use of probiotics has increased in recent years, aiming to restore the balance of gut microbiota for the benefit of the host (29). The role of miRNAs in modulating gut microbiota underlined their possible implication in the molecular mechanism of action of probiotics (13, 14). This review summarizes the most important current knowledge about the interplay between gut microbiota and miRNAs in development of CVDs. Firstly, the importance of miRNAs-gut microbiota relationship is highlighted. Moreover, the role of the gut microbiota and miRNAs in CVDs was discussed in detail. The current therapeutic strategies and the importance of discovering new treatment options for maintaining the balance of gut microbiota and, therefore, the health of the host are also underlined.

## miRNAs and Gut Microbiota

miRNAs are small non-coding RNAs that consist of 18–23 nucleotides in length. RNA polymerase II transcribes miRNA in the nucleus, producing pre-miRNA. It is then carried to the cytoplasm, processed into mature forms and loaded onto the mi-RNA-induced silencing complex (miRISC). By hybridizing with the 3′ untranslated region of the target gene, it promotes messenger ribonucleic acid (mRNA) degradation or inhibit translation (12, 30, 31).

Many studies have highlighted the connection between miRNAs and gut microbiota, especially in case of dysbiosis, which is leading to dysfunction of the intestinal epithelial barrier and inflammatory response (32). miRNAs are particularly involved in the regulation of the intestinal epithelial cells (IEC) (33).

It has been shown that expression of miRNAs is different between small and large IECs (34) and that miRNA-microbiota abundance has an inverse correlation (13). IECs can produce fecal miRNAs that target bacterial mRNA, controlling the destruction or dysregulation of their expression (13). In the dendritic cells of mice suffering from colitis, commensal bacteria can down-regulate the expression of miRNAs, for example miR-10a (13). Studies on mice have shown that expression of miRNAs is also different between colonized mice and germ-free mice (35), influencing intestinal-related diseases. Some studies (36–38) have shown that fecal miRNAs have the ability to influence the gut microbiota composition.

miR-375 has an important role in the production of the mucus layer in epithelial cells and influences the proliferation of stem cells in the intestinal epithelium (39, 40). The suppression of miR-381-3p is leading to proliferation of the IECs mediated by nuclear receptor-related protein, while the intestinal barrier function is being enhanced (41).

Gut microbiota is represented by the multitude of microbes within the human intestinal tract, while gut microbiome consists of the totality of their genetic properties. The gut microbiota can produce biologically active metabolites, which can further influence host physiological processes. Alterations in the microbial population can lead to multiple diseases, including CVDs (42, 43).

Dysbiosis, intestinal inflammation and altered gut barrier can result in high concentrations of bacterial structural elements and microbial metabolites in the circulation, including trimethylamine N-oxide (TMAO) and short-chain fatty acids, which can further favor the evolution of CVDs (44). Low concentrations of microbes which produce butyrate have been associated with heart failure and coronary artery disease (45).

**Figure 1:**
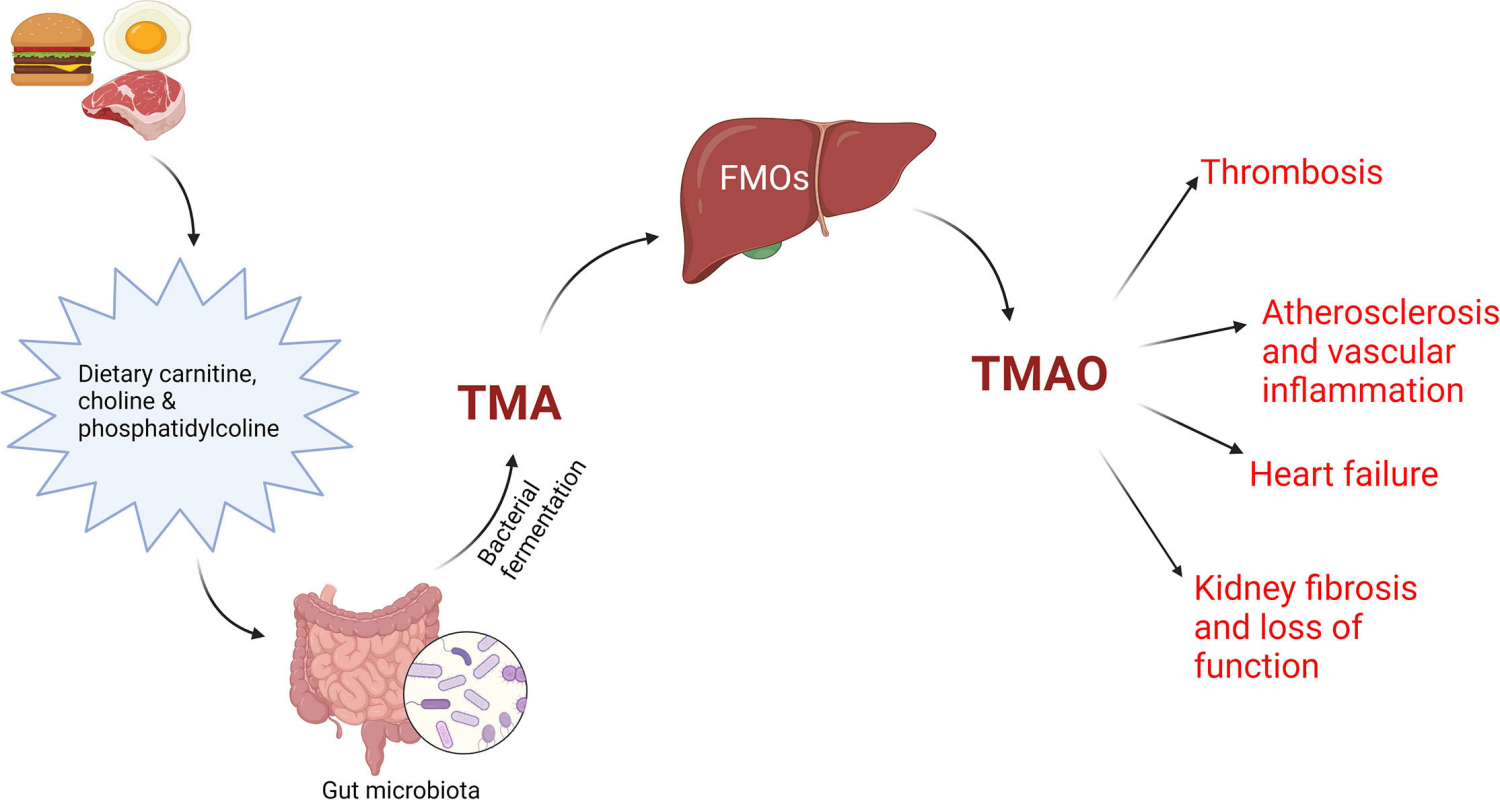
TMAO pathway. Dietary precursors, like carnitine, choline and phosphatidylcholine are metabolized by gut microbiota into trimethylamine (TMA), which will interact with flavin monooxygenases (FMOs) in the liver, producing trimethylamine N-oxide (TMAO), an important factor in the pathogenesis of CVD. Created with BioRender.com (Last accessed on 16 January 2022).

Blood levels of TMAO and phenylacetylglutamine, gut microbiota-dependent metabolites, have been linked to incident CVDs risks after several clinical studies. Phenylacetylglutamine has the potential to favor negative cardiovascular phenotypes in hosts, by acting on adrenergic receptors. TMAO is a metabolite frequently found in Western diets, rich in lecithin, choline (46), phosphatidylcholine (46, 47) and carnitine (48), which predicted the cardiovascular risk in some clinical studies and amplified the atherosclerotic process in animal models (43, 46). In many studies, a plasma value of TMAO over 6 μM anticipated a high risk of adverse cardiac events (49). The TMAO pathway and its effect on CVDs are illustrated in Figure 1.

Circulating TMAO levels are influenced by diet, gut microbial composition and renal function. Several studies indicate a dose-dependent association between meat ingestion and cardiovascular risk and mortality (50). Circulating TMAO concentrations have been linked to CVDs and anticipated prognosis in peripheral artery disease (51), coronary artery disease (52), acute coronary syndromes (52–54) and heart failure (55–59).

The human gut hosts about 1,000 microbial species (60), with two important phyla, *Bacteroidetes* and *Firmicutes*, representing the majority, along with *Actinobacteria, Cyanobacteria, Fusobacteria, Proteobacteria* and *Verrucomicrobia* (60–62). When disturbances of the immune system occur, levels of facultative anaerobes rise and change the ratio of *Bacteroidetes* to *Firmicutes* phyla, with a significant decline in Bifidobacteria (63).

Liu et al. (38) found that a specific subtype of miRNA has a specific role on gut microbiota: miR-515-5p can influence the growth of *Fusobacterium nucleatum* and miR-1226-5p can stimulate the growth of *Escherichia coli*. Other studies have underlined that gut microbiota and pathogenic microorganisms have different effects on different types of miRNAs, for example miR-203, miR-483-3p and miR-595 are inhibited by *enteropathogenic Escherichia Coli*, leading to an intestinal inflammatory status by disrupting the tight junction proteins of the intestinal epithelium (64). miR-182, miR-503 and miR-17-92 can increase the risk of malignancy in correlation with the existence of several taxa, like *Proteobacteria, Firmicutes* and *Bacteroidetes* (13, 65). Bitar et al. found miR-155 and miR-144a in IECs of patients with *Vibrio cholerae* infection in the acute stage, inhibiting the immune system at intestinal level and lowering cytokine secretion (39). Li X et al. have shown that miR-301b can inhibit the secretion of anti-inflammatory cytokines IL-4 and TGF-β1 in patients with *Pseudomonas aeruginosa* infection (66). miR-143-3p increases the risk of gastric malignancy in *Helicobacter pylori* positive patients by inhibiting AKT2, a gene that stimulates cell apoptosis and inhibits tumor growth, migration, and invasion (67). Also, pathogenic microorganisms have the ability to influence miRNAs in hosts. For example, miR-23a-5p can inhibit *Mycobacterium tuberculosis*-induced autophagy in macrophages, assuring bacterial survival (68).

Recently, studies have underlined the involvement of miRNAs in intercellular communication through a RNA delivery system controlled by EVs (13). Gut microbiota can produce and secrete EVs, especially exosomes, which carry genetic material. miRNAs derived from exosomes are involved especially in the communication between dendritic cells, influencing gene expression (19, 69, 70). Dai et al. showed that the downregulation of some bacterial carriers, *via* miR-193a-3p, is reducing colonic inflammation (71).

By influencing expression of miRNAs in other organs, inflammation of the gut can lead to many diseases, including CVDs (26). For example, gut microbiota can stimulate miR-204, *via* the signal transducer and activator of transcription three signaling pathway, inhibiting its target gene, Sirtuin 1. This will lead to a lower endothelium-dependent vasorelaxation and atherosclerosis (23). Moreover, studies have shown that anthocyanins reduce atherosclerosis and stimulate cholesterol efflux from macrophages. Gut microbiota can metabolize cyanidin-3 to 0-β-glucoside and produce protocatechuic acid (PCA), the most important constituent of anthocyanins. PCA can inhibit the expression of miR-10b in macrophages and reduce the atherosclerotic process, by stimulating a reverse cholesterol transport (22, 24).

## Cardiovascular Diseases

Since their discovery in 1993 by Lee et al. (72, 73), miRNAs have been known as important regulators of various biological functions, also being involved in the pathogenesis of CVDs (74, 75). miRNAs are found in cardiac tissue through all phases of organ development. miR-21, miR-29a, miR-129, miR-210, miR-211, miR-320, miR-423 and let-7c have been identified in fetal heart tissue (74). The most abundant miRNAs in cardiac tissue are miR-1, let-7, miR-133, miR-126-3p, miR-30c and miR-26a (76), while in arteries the most frequent miRNAs are miR-145, let-7, miR-125b, miR-125a, miR-23 and miR-143 (77). In the human heart, several other miRNAs have been often identified: miR-16, miR-100, miR-125 a/b, miR-145, miR-195, miR-199^*^, miR-20a/b, miR-21, miR-24, miR-23, miR-29a/b, miR-27a/b, miR-30a/b/c, miR-92a/b and miR-99 (78, 79).

**Figure 2:**
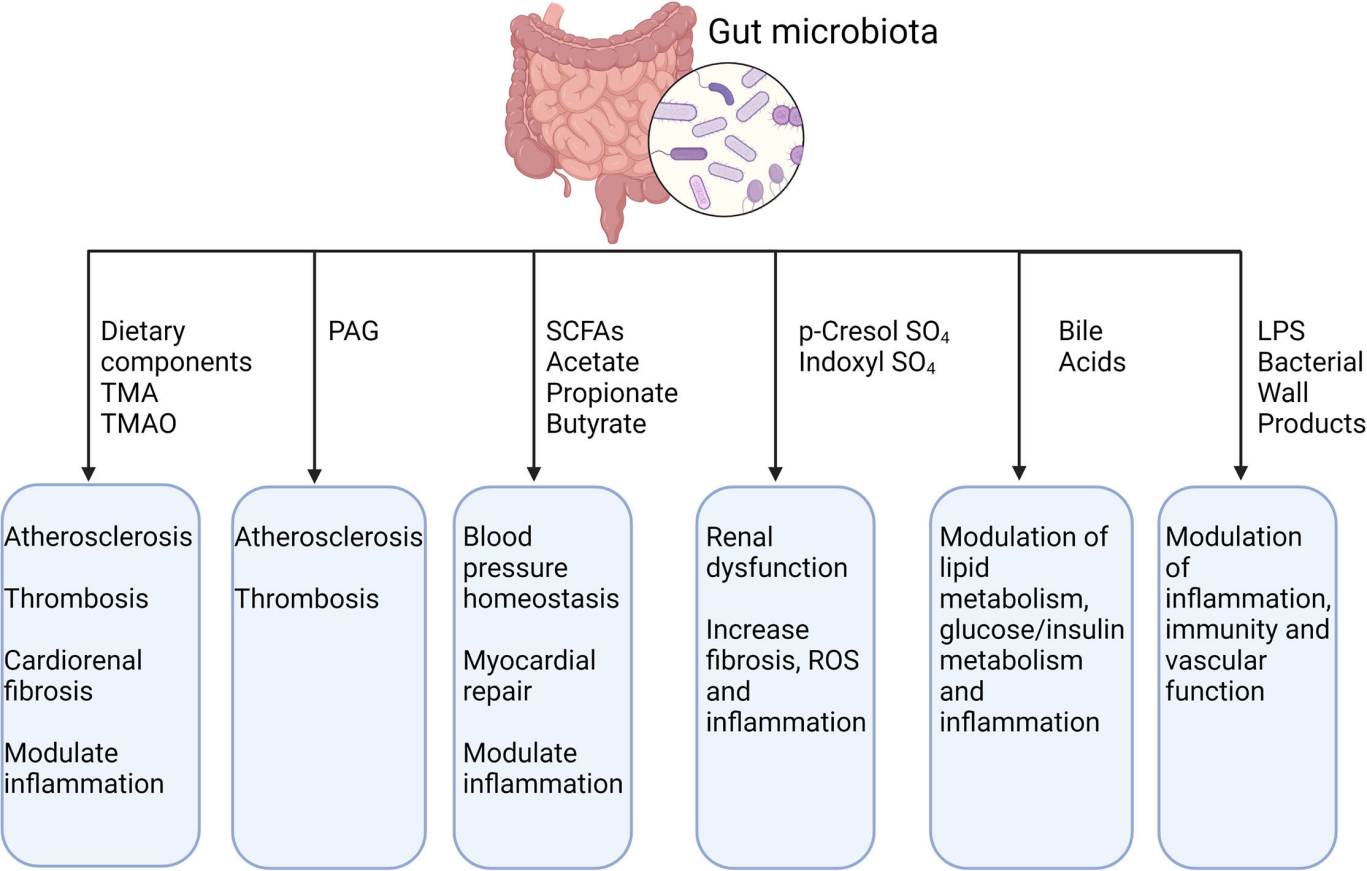
Gut microbiota, its metabolites and their effect on CVDs. TMA, trimethylamine; TMAO, trimethylamine N-oxide; PAG, phenylacetylglutamine; SCFAs, short-chain fatty acid; LPS, lipopolysaccharide. Created with BioRender.com (Last accessed on 16 January 2022).

Gut microbiota and its metabolites influence many cardiovascular phenotypes, such as atherosclerosis, platelet reactivity, thrombosis, hypertension, lipid and glucose metabolism and vascular inflammation, as we summarized in Figure 2.

### Atherosclerosis

Atherosclerosis is a complex progressive process, involving dysfunction of endothelial cells, vascular smooth muscle cells differentiation, infiltration with inflammatory cells and subendothelial accumulation of lipids (74, 80).

miR-126-5p maintains a proliferative reserve in endothelial cells by inhibiting the Notch1 inhibitor delta-like homolog 1. Decreased levels of miR-126-5p lower the capacity of proliferation of endothelial cells, losing the ability to protect against the formation of atherosclerotic lesions. A novel possible curative perspective could be the administration of miR-126-5p, which improved the proliferation of endothelial cells in certain locations and restricted atherosclerosis, according to Schober et al. (81).

miR-155, a possible prognostic factor and therapeutic target, represses anti-inflammatory signaling in macrophages. Low concentrations of miR-155 are found during the reversion of atherosclerosis *in vivo*. Furthermore, miR-155 is found in high concentrations in urinary EVs in patients with unstable coronary artery disease (82). Moreover, Zhao et al. have shown that in ovariectomized mice with consecutive estrogen related metabolic syndrome, changes of lumen diameters in common carotid artery, left ventricle ejection fraction and fractional shortening was significantly lower than in sham-operated group, but they registered higher common carotid artery thickness. They observed that miR-155 and let-7g were upregulated in the intestinal epithelium and feces and endocytosed by bacteria, modulating the microbiota and cardiovascular function in the same way as in ovariectomized mice (15).

**Figure 3:**
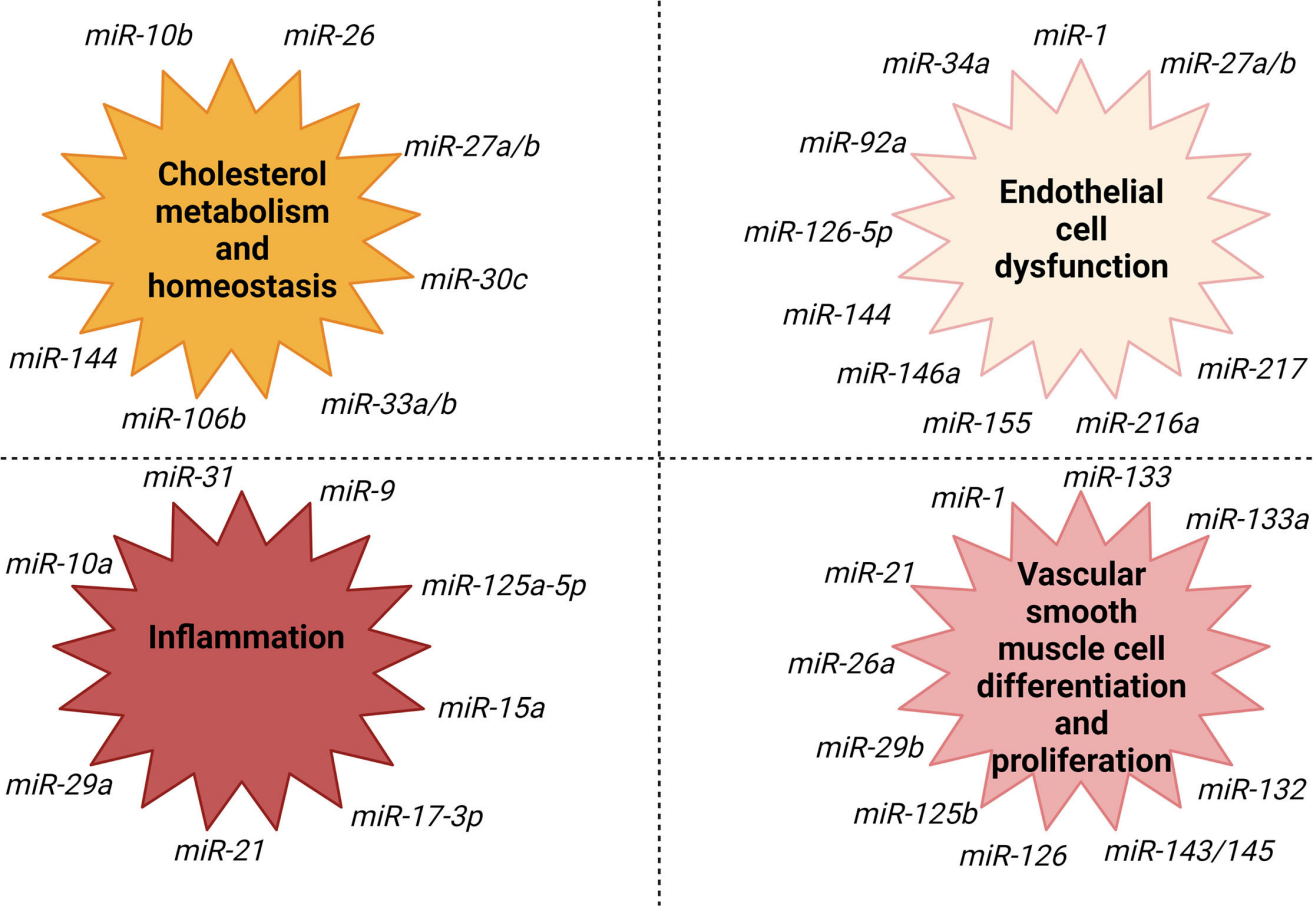
miRNAs association with fundamental processes involved in the evolution of atherosclerosis. Created with BioRender.com (Last accessed on 16 January 2022).

Another important step in atherosclerosis is vascular smooth muscle cells migration from the media to the intima as a reaction to infiltration with inflammatory cells (74). Some miRNAs are involved in this step of the process. miR-145 is vastly expressed in vascular smooth muscle cells (83). miR-143/145 complex has an important role in influencing the differentiation and plasticity of vascular smooth muscle cells (74, 83, 84). The development of the atherosclerotic plaques was diminished, while the plaque stability was higher in ApoE knockout mice that received vascular smooth muscle cells-targeted miR-145 therapy (83). In Figure 3 are illustrated the most important processes involved in the evolution of atherosclerosis and their association with different subtypes of miRNAs.

Any infectious status will impact atherosclerosis, as it is an inflammatory condition. There are two major mechanisms involved: direct infection of the blood vessel wall or through an indirect mechanism, *via* infections of other sites, generating proinflammatory mediators within a systemic immune response, which will further influence the growth of the atherosclerotic plaque (85). A high risk of CVDs has been associated with the presence of C*hlamydophila pneumoniae, Porphyromonas gingivalis, Helicobacter pylori*, Influenza A virus, Hepatitis C virus, cytomegalovirus and human immunodeficiency virus (86).

TMAO is an intestinal metabolite of choline and phosphatidylcholine. Flavin monooxygenases 3 is a regulator of TMAO synthesis, being adjusted by farnesoid X receptor (FXR), whose levels are increased by bile acids. TMAO contributes to atherogenesis by blocking reverse cholesterol transport, leading to low cholesterol clearance from peripheral macrophages. TMAO also alters the protective properties of high-density lipoproteins (44). TMAO increases platelet hyperreactivity by amplifying stimulus-dependent release of calcium from intracellular sites, elevating the risk of thrombosis (87).

Bile acids influence the cardiac function through different mechanisms. They interact with plasma membrane G-protein-coupled receptors and nuclear receptors (FXR) and can modify the contractility of cardiomyocytes and the electrical properties (reducing heart rate by influencing channel conduction and calcium transport). Bile acids also play important roles in lipid metabolism, plaque formation, vasodilation and neovascularization of wounded organs. Conditions associated with high serum concentrations of bile acids, such as advanced liver disease, obstructive jaundice, intrahepatic cholestasis, are associated with abnormal vascular dynamics (88).

### Hypertension

The role of miRNAs in hypertension was underlined by several studies and experimental results, especially by interfering with the renin-angiotensin-aldosterone system (89).

miRNAs can regulate renin expression, according to Marques et al. Several miRNAs found in human hypertensive kidneys (hsa-let-7c, hsa-miR-181a, hsa-miR-663) bind to the 3′ untranslated region and regulate AIFM1 (apoptosis-inducing factor mitochondrion-associated 1), APOE (apolipoprotein E), REN (renin) and NR4A2 (nuclear receptor subfamily four group A member 2) mRNA (90). Low levels of hsa-miR-181a and hsa-miR-663 in hypertensive kidneys can be an explanation of higher renin mRNA (91). REN mRNA overexpression and the downregulation of two miRNAs (hsa-miR-181 and hsa-miR-663) which influence REN mRNA levels indicate the involvement of renin in the etiology of hypertension (91).

In the renal medulla, SIK1 (salt-inducible kinase 1) can elevate blood pressure by intensifying active cell sodium transport, as a response to angiotensin II. In hypertensive animal subjects, the activity of SIK1 is increased in the cells from the proximal tubule. As a response to angiotensin II, SIK1 can activate NR4A2 (from the nuclear transcription factor family of NR4A), which has a role in cytokine modulation and may therefore lead to inflammation in the renal medulla in hypertensive subjects (91).

miR-155 targets hypertension-associated SNP (rs5186) in the angiotensin II type 1 receptor 3′ untranslated region. High levels of angiotensin II type 1 receptor in young hypertensive homozygous subjects for the rs5186 C allele correlate positively with blood pressure and negatively with hsa-miR-155 expression (91).

miRNAs regulate gene expressions in cellular responses to vasopressors, such as angiotensin II. miR-132 and miR-212 are transcribed together, regulated by cAMP response element binding protein, which is an angiotensin II-controlled gene (91).

miR-132 and miR-212 were found to be up-regulated in the left ventricle, aorta and kidneys of animals who received infusion with angiotensin-II for a period of 10 days and down-regulated at the level of the internal mammary artery of patients who received angiotensin-II receptor type 1 blockers. This indicates that miR-132 and miR-212 are associated with angiotensin II-induced Gαq-signaling pathway, generating hypertension (74, 92). Eskildsen et al. concluded that miR-132 and miR-212, found in human arteries from patients who underwent bypass surgeries and who were treated with angiotensin II receptor 1 blockers, were in low concentrations, compared to patients treated with beta blockers, which had no effect (91). As a conclusion, miR-132 and miR-212 are involved in angiotensin II induced hypertension (91).

miR-130 and miR-195 in the blood were correlated with hypertension in a study conducted by Karolina et al. (92). miR-197, miR-23a and miR-509-5p contribute to dyslipidemia in metabolic syndrome. miR-150, miR-192, miR-27a, miR-320a and miR-375 were up-regulated in subjects with metabolic syndrome and type 2 diabetes (93).

Santovito et al. concluded that miR-145 was overexpressed in atherosclerotic plaques of hypertensive patients compared to the control group. Downregulation of miR-145 was correlated with vascular smooth muscle cell dedifferentiation, essential for atherosclerosis. Subjects treated with angiotensin II receptor blockers were associated with a maximum elevation of miR-145 concentrations, but this concept did not have statistical significance due to the small sample size (93).

### Heart Failure

Cardiac structural modifications allow the heart to adapt to various hemodynamic conditions. Chronic triggering of cardiac remodeling (myocyte hypertrophy, fibrosis) can lead to cardiomyopathies and heart failure (74).

miR-1 and miR-133, muscle-specific miRNAs, expressed in cardiac and skeletal muscles, are able to influence the differentiation and proliferation of muscle cells. They are the most frequent miRNAs in the cardiac tissue (76, 78, 94).

The role of miRNAs in the physiological functioning of the adult heart has been validated with Dicer (RNase enzyme involved in miRNA processing, cleaving pre-miRNAs into miRNAs duplexes) deletion. This deletion led to dilation and fibrosis of both ventricles, hypertrophy of cardiac myocytes, initiation of fetal transcriptome programme and sudden death, underlining the influence of miRNAs on cardiac remodeling (miR-133, miR-1) (74).

In both animal and human models with cardiac hypertrophy, low levels of miR-133 and miR-1, that are included in the same transcriptional unit, were noticed. They are considered key regulators of cardiac hypertrophy. *In vitro* overexpression of miR-133 and miR-1 suppressed cardiac hypertrophy, according to Carè et al. *In vivo* repression of miR-133 by using antagomir (miRNA antagonist) infusion led to important and prolonged cardiac hypertrophy. Specific targets of miR-133 are: RhoA, a GDP-GTP exchange protein controlling cardiac hypertrophy; Cdc42, a signal transduction kinase involved in hypertrophy, and Nelf-A/WHSC2, a nuclear factor with role in cardiogenesis (95).

miRNAs as new potential therapeutic targets started to be investigated since miRNAs that inhibit contractility are upregulated in heart failure patients (96). High levels of endogenous miR-25 reduced the cardiac function, while antagomiRs targeting miR-25 restored it, with consequential enhanced survival, compared to the control group in an animal model study. Altered calcium uptake during excitation-contraction coupling in cardiomyocytes comes from low expression and activity of SERCA2a (a ATPase involved in calcium transport), which is a feature of heart failure pathophysiological events (96, 97).

According to Gurha et al., a targeted deletion of miRNA-22 favored stress-induced cardiac dilation and contractile impairment. miR-22-deficient animal models had altered inotropic and lusitropic responses to acute stress tests with dobutamine. Genetic ablation of miR-22 resulted in low cardiac expression of SERCA2a. Muscle-restricted genes encrypting proteins in the proximity of the cardiac Z disk/titin cytoskeleton were also a result of the ablation of miR-22. In the absence of miR-22, there was an elevated expression of transcriptional/translational repressor purine-rich element binding protein B, which withstands control of sarcomeric/cardiac expression by serum response factor (98).

However, miRNAs can be regarded as paracrine signaling mediators. Cardiac fibroblasts secrete exosomes which contain miRNAs, “star” miRNAs (miRNA passenger strands, strands that are usually broken down within a cell) more precisely (99). Fibroblast-derived miR-21 can function as an essential paracrine signaling mediator in cardiomyocyte hypertrophy, making miR-21 a new possible target in heart failure treatment (99). Moreover, Thum et al. stated that miRNA-21 can contribute to myocardial disease by stimulating the MAP kinase signaling in fibroblasts, with effects on global cardiac structure and function. In the fibroblasts of failing hearts, miR-21 levels are higher, amplifying the ERK-MAP kinase activity by blocking sprouty homolog 1 (Spry 1) and modulating fibroblast survival and growth factor secretion (with impact on interstitial fibrosis and cardiac hypertrophy). Suppressing miR-21 using targeted antagomiR in animal models with induced pressure overload disease diminished the activity of cardiac ERK-MAP kinase, suppressed interstitial fibrosis and decreased cardiac dysfunction (100).

High levels of miRNAs that suppress SERCA2a can be found in heart failure cases, which can further lead to weakened cardiac function. In conclusion, miRNAs that downregulate SERCA2a can be in elevated levels in heart failure and consequently damage cardiac function (96).

In patients with heart failure, the gut microbiome is characterized by low levels of Ruminococcaceae family, of *Blautia* (Lachnospiracea family) (101) and of *Faecalibacterium prausnitzii* (102). Relative diminished populations of *Eubacterium rectale* and *Dorea longicatena* (Lachnospiraceae family) and *Faecalibacterium* (Ruminococcaceae family) were also observed in older patients (103). These results indicate decreased levels of butyrate-producing microorganisms (belonging to Lachnospiraceae or Ruminococcaceae families) in heart failure patients (45). Circulating TMAO levels have predicted prognosis in patients with peripheral artery disease (51), coronary artery disease (52), acute coronary syndrome (52–54) and heart failure (55–59).

### Arrythmias

In cardiac rhythm abnormalities, several miRNAs were noted to be involved (104).

miRNAs can influence the characteristics of cardiac physiology and excitability (104). The focus of several studies was on miR-1 and miR-133, as they are some of the most frequently expressed miRNAs in cardiac tissue (79, 94).

For instance, in patients with atrial fibrillation, miR-1 concentrations are severely low in the atrial tissue. Overexpression of miR-328 amplified the vulnerability to develop atrial fibrillation, decreased L-type calcium current and reduced the atrial action potential duration (105). The use of antagomiR for miR-328 overturned these conditions. miR-1 has been involved in the adjustment of various calcium-handling proteins (calmodulin, phospholamban, Na^+^/Ca^2+^ exchanger, sorcin, junction, triadin), leading to shorter refractory period of sarcoplasmic reticulum Ca^2+^ release (104) and has been connected to ventricular arrhythmias (74).

There are several miRNAs involved in the electrical and structural changes in atrial fibrillation (Table 1).

**Table 1:** miRNAs involved in the electrical and structural changes in atrial fibrillation.

**miRNAs**	**Target genes**	**Effect**	**Roles**	**References**
miR-1	KCNJ2	Increased I_k1_	Down-regulation in AF	(106, 107)
	GJA1/ connexin43	Slower conduction	^*^Atrial electrical remodeling	(108)
	HCN2	Amplified automaticity	^*^Ectopic activity	(109)
	HSP60, HSP70	Favors apoptosis	^*^Atrial structural remodeling	(110)
miR-21	Spry1,	Up-regulated	Inhibits fibroblast proliferation	(111)
	PDCD4		Anti-apoptosis	(78, 112)
miR-26	KCNJ2/Kir21	Down-regulated	Increases I_k1_	(113, 114)
		Atrial electrical remodeling	APD shortening	(79, 114)
miR-29	Fibrillin Collagen 1A1, Collagen 3A1	Down-regulated	Increased fibrosis	(115)
	Mcl-2	^*^atrial structural remodeling	Pro-apoptosis	(100)
miR-30	CTGF	Down-regulated	Increased fibrosis Matrix remodeling	(110, 116)
miR-133	CTGF	Down-regulated	Increased fibrosis	(116, 117)
		^*^Ectopic activity	Matrix remodeling	
	TGF-b1 TGFbRII	Atrial structural remodeling	Anti-fibrosis	(118)
	HCN2 HCN4	Enhanced automaticity	Down-regulation in AF	(109)
	Caspase 9	Anti-apoptosis	(110)	
miR-208	THRAP1	^*^Pro-fibrosis	Atrial structural remodeling	(119)
miR-328	Up-regulated	CACNB1, CACNA1C	Shorter atrial action potential duration	(105)
		Atrial electrical remodeling	APD shortening	(120)
miR-499	KCNN3	Up-regulated	Altered conduction	(121)
miR-590	TGF-b1 TGFbRII	Down-regulated in AF	Anti-fibrosis Atrial structural remodeling	(118)

*AF, atrial fibrillation; KCNJ2, K+ inwardly-rectifying channel, subfamily J, member 2; Ik1, inward-rectifier K+ current; GJA1, Gap junction alpha1 protein; HCN, hyperpolarization-activated cAMP-gated non-selective cation channel; HSP, heat shock protein; SPRY1, sprouty homolog 1; PDCD4, programmed cell death 4; APD, atrial potential duration; Mcl-2, myeloid cell leukemia 2 (an anti-apoptotic Bcl-2 family member); CTGF, connective tissue growth factor; TGF b, transforming growth factor b; THRAP1, co-regulator of the thyroid hormone receptor; CACNB1, cardiac IcaL channel beta1 subunit; CACNA1C, cardiac IcaL channel alpha1c subunit; KCNN3, K+ intermediate/small conductance Ca2+ activated channel*;

* *speculations, which need further experimental studies; A-ER, atrial electrical remodeling; A-SR, atrial structural remodeling*.

### Coronary Artery Disease

A new possible therapeutic target for ischemic heart disease was suggested by the results communicated by Ren et al. Overexpression of miR-320 promoted the apoptosis of cardiac cells and expanded the infarction size in ischemia-reperfusion cases *in vivo* and *ex vivo*, compared to wild-type controls. The heat-shock protein 20 (Hsp20), a cardioprotective protein, is a significant target for miR-320. miR-320 acts as a negative regulator of Hsp20 translation. During the ischemia-reperfusion *in vivo* and *ex vivo*, the expression of Hsp20 was notably higher after 1 h from reperfusion and 24 h from reperfusion (*in vivo*). This was negatively correlated with low expression of miR-320 *in vivo* and *ex vivo* (in cardiomyocytes). To sum up, expression of miR-320 can be correlated with Hsp20 levels in the cardiac tissue, while miR-320 can reliably suppress expression of Hsp20 *in vivo* and *ex vivo* (122).

As miRNAs can be found in the bloodstream, they can be regarded as new biomarkers, especially for myocardial infarction and heart failure (74). The role of cardiac troponins in the diagnosis of acute coronary syndromes is not likely to be outperformed, however miRNAs may be relevant in completing existing prediction patterns and as prognostic markers after acute coronary syndromes (74). Devaux et al. investigated the concentrations of some miRNAs in a large group of patients with acute chest pain. Levels of miR-208b, miR-499 and miR-320a were significantly elevated in patients with acute myocardial infarction in comparison with other diagnoses. However, no miRNA was able to surpass high sensitive troponin T or amplify their diagnostic capacity. Moreover, none of the miRNAs had an important prognosis accuracy (74, 123).

Circulating miRNAs were assessed for the potential role as circulating biomarkers. miR-126 had a positive association with incident myocardial infarction, while miR-223 and miR-197 were inversely related to the risk of myocardial infarction (124).

**Figure 4:**
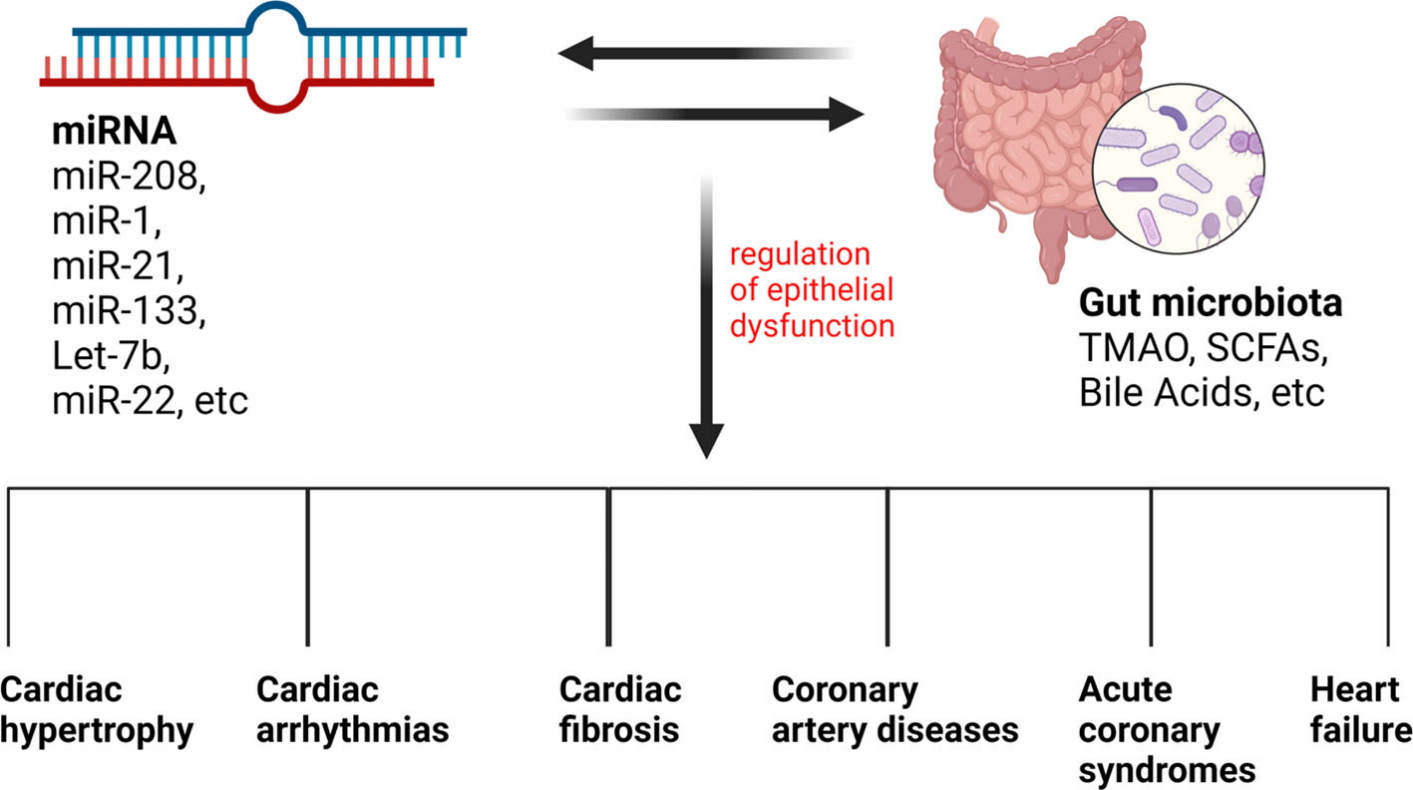
The relationship between gut microbiota, miRNAs, and CVDs. Different types of miRNAs and gut microbiota metabolites can influence each other, influencing the regulation of epithelial dysfunction and leading to many CVDs.

As a conclusion, we listed the miRNAs involved in different CVDs in Table 2 and illustrated the relationship between gut microbiota, miRNAs and CVDs in Figure 4.

**Table 2:** The regulation of miRNA in different CVDs.

**Cardiovascular diseases**	**miRNA**	
	**Up-regulation**	**Down-regulation**
Cardiac hypertrophy	miR-208, miR-21, miR-29, miR-18b, miR-199, miR-23, miR-22 (125) miR-195 (126)	miR-1, miR-133, (125) miR-9 (127)
Cardiac arrhythmias	miR-1, miR-133, miR-708-5p, miR-217-5p, miR-208 (125)	miR-499-5p (125)
Cardiac fibrosis	miR-21, miR-15 (125)	miR-133, miR-29, miR-26a, miR-24, miR-590 (125)
Coronary artery diseases	miR-1, miR-21, miR-33, miR-208, (125) miR-25, miR-92a, miR-106b, miR-122, miR-133a, miR-135a, miR-140-3p, miR-146a, miR-155, miR-182, miR-186, miR-370, miR-451, miR-490-3p (81)	miR-133, miR-126-3p, miR-195, miR-145, miR-155, miR-17, miR-93-5p (125) miR-29a, miR-31, miR-125b, miR-147, miR-181a, miR-214, miR-320b (81)
Acute coronary syndromes	miR-1, miR-21, miR-30a, miR-30c, miR-34a, miR-122, miR-126, miR-133a, miR-133b, miR-134, miR-145, miR-146a, miR-155, miR-186, miR-195, miR-198, miR-199, miR-208, miR-223, miR-320a, miR-328, miR-370, miR-423-5p, miR-433, miR-485-3p, miR-499 (81)	Let-7b, miR-29a, miR-122, miR-125b, miR-126, miR-155, miR-223, miR-320b, miR-375, miR-663b, miR-1291 (81)
Heart failure	miR-199b, miR-24, miR-208, miR-125, miR-195, miR-214, miR-423-5p, miR-320a, miR-22, miR-92b, miR-122, miR-650, miR-662, miR-1228, miR-100, miR-342 (125) miR-18b, miR-21, miR-29b, miR-129-5p, miR-133a, miR-142-3p, miR-200b, miR-210, miR-499, miR-519e, miR-520d-5p, miR-622, miR-675, miR-1254 (81)	miR-126, miR-133, miR-1, miR-107, miR-3175, miR-583, miR-29b (125) miR-30b, miR-103, miR-125b, miR-139, miR-142-3p, miR-142-5p, miR-342-3p, miR-497 (81)

## Therapeutic Strategies

The concept of therapies based on miRNAs is gaining more and more attention, as synthetic antagonists of miRNAs are studied already in the treatment of various diseases, such as hepatitis C virus infection, dyslipidemia or heart failure (74). Cardiovascular afflictions are no exception, progress and research being made in this field as well, for example for dyslipidemia or heart failure (74).

Targeting the gut microbiome in patients with CVDs may improve clinical outcomes and prognosis. Proper fiber intake may reduce low-density lipoprotein (LDL) concentrations, lowering the risk of CVDs and coronary heart disease. Water soluble fibers, such as beta-glucan, psyllium, pectin, guar gum were the most successful in lowering serum LDL cholesterol levels, while not altering high density lipoprotein (HDL) concentrations (128). Whole grain consumption has been inversely associated with metabolic syndrome (*p* = 0.005) and mortality from cardiovascular conditions (*p* = 0.04) (129).

**Figure 5:**
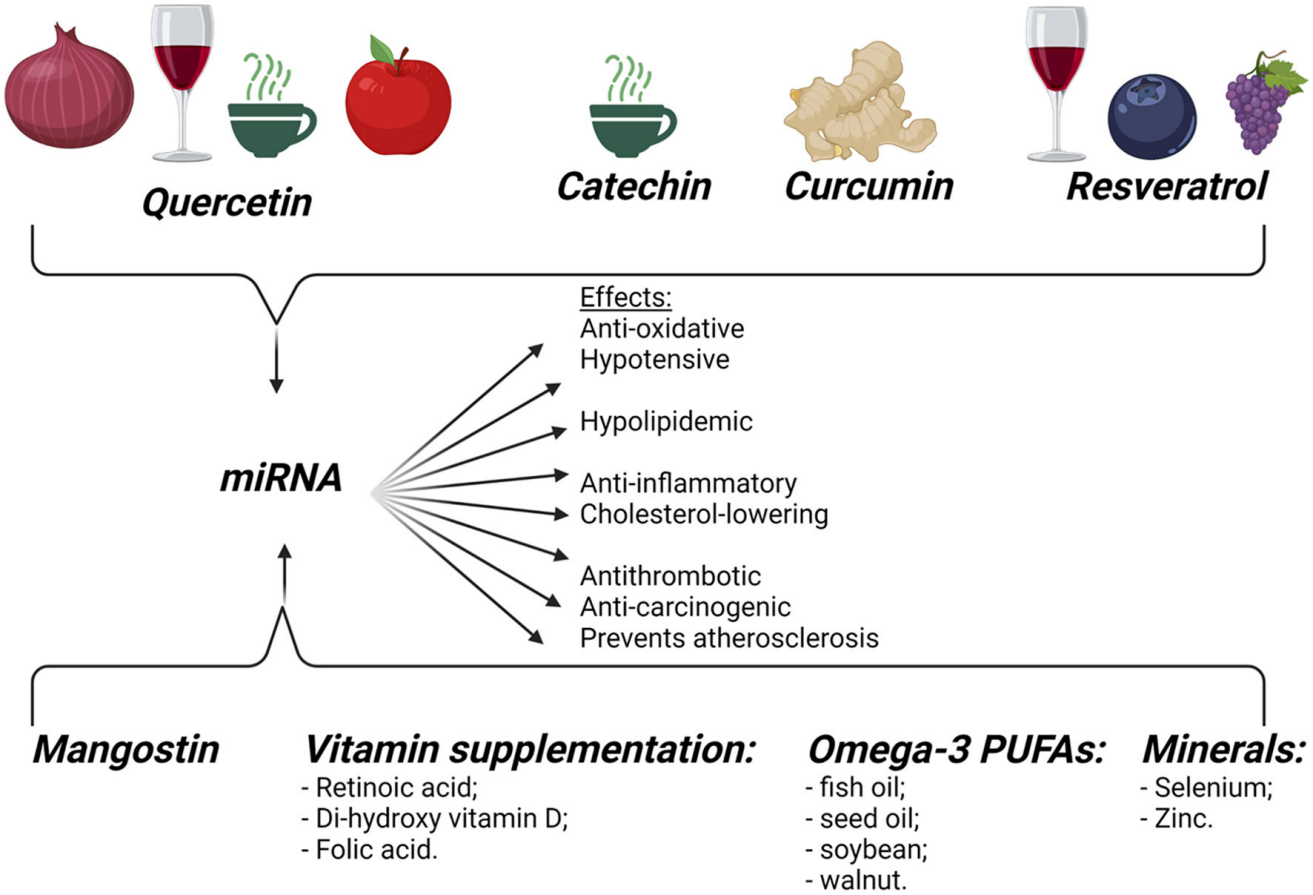
Dietary influence on miRNAs. Dietary food components can influence gene expression, modifying the pathogenesis of different diseases, like cancer, obesity, and CVDs. Created with BioRender.com (Last accessed on 12 January 2022).

The influence of diet modifications on miRNAs is illustrated in Figure 5.

Many studies have underlined the positive effects of the probiotics on gut homeostasis. As we mentioned above, miRNAs also influence the gut microbiota. Considering that, recent studies have tried to understand and demonstrate the hypothesis that miRNAs could have an important role in how probiotics regulate gut homeostasis and influence gastrointestinal conditions or extra-intestinal disorders, including CVDs (27).

The beneficial effects of the probiotics are well known, as they produce bacteriocins, lower pH and compete with pathogenic microorganisms, in order to restore and stabilize the gut microbiota (130, 131). For example, organic acids (produced by *Lactobacillus* and *Bifidobacteria* probiotic strains) and bacteriocins can lower intestinal pH and inhibit the growth of the pathogenic bacteria (132). Also, the same probiotic bacteria mentioned above can improve the intestinal barrier function, by upregulating the genes that are controlling the mucus secretion (133, 134) and can influence the IECs proliferation (135).

Due to recent discoveries, it is now clear that gut microbiota is very responsive to the influence of probiotics, regulating general health and organic disorders, including CVDs, especially atherosclerosis and its acute consequences (coronary heart disease and stroke) (9) and hypertension (26). Some studies have shown that probiotics can modify the levels of metabolites, DNA, and components of the immune system, can activate immune cells, stimulate the production of immunoglobulins and regulate cytokines, demonstrating anti-inflammatory and immune effects in the host (134, 136). However, the connection between probiotics, miRNA, gut microbiota and organic diseases is still not well known, being a relatively novel field of research that needs further investigations.

Another therapeutic option, which seems to be more and more studied recently, is represented by fecal miRNAs and their influence on gut microbiota. According to Liu et al., fecal miRNAs produced by IECs and Hopx+ cells can cross the bacterial wall and control specific gene transcripts, influencing their growth. Mice with lower levels of IEC-derived miRNAs develop dysbiosis and fecal miRNAs transplantation can reestablish the microbiota composition in these cases (26, 38).

## Discussions

Cardiovascular diseases are the leading cause of mortality worldwide. There is constant emerging evidence regarding new possible interactions between miRNAs, gut microbiota and cardiovascular diseases. miRNAs may represent important diagnostic and/or prognostic markers, nevertheless large studies are needed to determine the suitable target population and to identify reliable, standardized and consistent methods for the quantification of circulating miRNAs. miRNA-based therapies in cardiovascular medicine need to assure a safe and targeted delivery in patients, constituting attractive new treatment options. Besides dietary interventions and probiotics, new possible therapeutic methods that may target the composition of gut microbiota for the treatment or prevention of cardiovascular diseases constitute a promising direction of investigation.

## Author Contributions

All authors listed have made a substantial, direct, and intellectual contribution to the work and approved it for publication.

## Conflict of Interest

The authors declare that the research was conducted in the absence of any commercial or financial relationships that could be construed as a potential conflict of interest.

## Publisher's Note

All claims expressed in this article are solely those of the authors and do not necessarily represent those of their affiliated organizations, or those of the publisher, the editors and the reviewers. Any product that may be evaluated in this article, or claim that may be made by its manufacturer, is not guaranteed or endorsed by the publisher.
